# Surface plasmon polariton–enhanced upconversion luminescence for biosensing applications

**DOI:** 10.1515/nanoph-2024-0247

**Published:** 2024-08-27

**Authors:** Duc Le, Marjut Kreivi, Sanna Aikio, Noora Heinilehto, Teemu Sipola, Jarno Petäjä, Tian-Long Guo, Matthieu Roussey, Jussi Hiltunen

**Affiliations:** 3259 Sensing Solutions, VTT Technical Research Centre of Finland, 90570 Oulu, Finland; Department of Physics and Mathematics, University of Eastern Finland, P.O. Box 111, FI-80101 Joensuu, Finland

**Keywords:** surface plasmon polariton, upconversion luminescence, gold grating, absorption enhancement, biosensing

## Abstract

Upconversion luminescence (UCL) has great potential for highly sensitive biosensing due to its unique wavelength shift properties. The main limitation of UCL is its low quantum efficiency, which is typically compensated using low-noise detectors and high-intensity excitation. In this work, we demonstrate surface plasmon polariton (SPP)-enhanced UCL for biosensing applications. SPPs are excited by using a gold grating. The gold grating is optimized to match the SPP resonance with the absorption wavelength of upconverting nanoparticles (UCNPs). Functionalized UCNPs conjugated with antibodies are immobilized on the surface of the fabricated gold grating. We achieve an UCL enhancement up to 65 times at low excitation power density. This enhancement results from the increase in the absorption cross section of UCNPs caused by the SPP coupling on the grating surface. Computationally, we investigated a slight quenching effect in the emission process with UCNPs near gold surfaces. The experimental observations were in good agreement with the simulation results. The work enables UCL-based assays with reduced excitation intensity that are needed, for example, in scanning-free imaging.

## 1 Introduction

Upconversion luminescence (UCL) is known as a large anti-Stokes emission where upconverting materials absorb two or more low-energy photons via metastable states and emit single higher-energy photons [1], [2], [3]. UCL can be excited with an incoherent source at low power density, which differentiates UCL from conventional nonlinear optical techniques [1], [2]. Upconverting nanoparticles (UCNPs) offer several unique properties for highly sensitive biosensing applications [4], [5], [6], [7]. In UCL measurements, autofluorescence and light scattering are avoided, thus enabling measurements without optical background interference [2], [5]. Furthermore, UCNPs emit light in different narrow bands with high color purity, which makes them ideal probes for multiplexed detection [8], [9]. Additionally, UCNPs have several advantages for bioassay developments, such as nonphotobleaching, nonblinking, low-toxicity, and photostability [4], [10]. Several groups have utilized UCNPs for highly sensitive biosensing. Farka et al. demonstrated that the UCNP-linked immunosorbent assay was about ten times more sensitive than the standard enzyme-linked immunosorbent assay (ELISA) [11]. Huang et al. quantified single small extracellular vesicles by using UCNPs with a limit of detection nearly three orders of magnitude lower than the ELISA [12]. Zhou et al. used UCNPs for the multiplexed simultaneous *in situ* detection of cancer biomarkers [13].

The main limitation of UCNPs is their relatively low quantum efficiency, which is smaller than 1 % [14]. One strategy for improving UCNP’s quantum efficiency is to modify intrinsic properties such as size, crystal host material, dopants, surface chemistry, and dispersion medium [3], [14]. Along with this strategy, engineering the external optical response of UCNPs using plasmonic nanoparticles or nanostructures can enhance UCL [15], [16], [17]. This method is called plasmon-enhanced UCL, aiming to improve either absorption or emission processes [18]. UCNPs have different excitation wavelengths and several emission wavelengths [3]. Here, we focus on the 976 nm excitation and 540 nm emission of NaYF4:Yb^3+^, Er^3+^, or Tm^3+^, which are commonly used in UCL measurements [14].

Plasmon-enhanced UCL relies on the excitation of either localized surface plasmon (LSP) or surface plasmon polariton (SPP). LSP-enhanced UCL has been studied by using plasmonic nanoparticles or nanostructures such as gold nanospheres [19], gold and silver nanosphere arrays [20], gold nanorods [21], gold nanorod array [22], [23], [24], gold–silver alloy island array [25], random silver nanowires network [26], and gold nanotrenches [27]. In these studies, the focus was on the enhancement of the absorption or emission process by matching the LSP resonance with the excitation wavelength or emission wavelength, respectively. For example, Ji et al. developed a novel LSP-enhanced UCNP sensor using plasmonic nanoparticles for supersensitive dithiothreitol detection [28]. They coated a UCL quenching layer around UCNPs, which decomposed in the presence of dithiothreitol. Lao et al. presented an LSP-enhanced UCL-based fluorescence resonance energy transfer (FRET) biosensor using gold nanoparticles for highly sensitive detection of SARS-CoV-2 viral RNA [29]. However, the major challenge of LSP-enhanced UCL is the precise control of the orientation and distance of the plasmonic surface with respect to the UCNPs [19], [24], [30]. This control is even more challenging in bioassay developments on plasmonic surfaces. Furthermore, the energy confinement of the LSP field is within a few tens of nanometers [24], [31], making it inappropriate for the bioassays of large analytes, e.g., exosomes ranging in size from 40 nm to 200 nm [12].

SPP-enhanced UCL offers some advantages for biosensing applications. The energy confinement of SPP field is within several hundreds of nanometers, about ten times higher than LSPs, from the plasmonic surface [31], [32]. Additionally, SPPs can be excited by using periodic nanostructures, offering tunable resonant wavelength by design and reproducible large-area fabrication [33]. Prymaczek et al. excited SPPs on one end of a long silver nanowire and observed UCL enhancement on the other end of the wire [34]. SPPs were excited by using a gold nanopillar array, leading to a few-fold enhancement in UCL [35]. Lu et al. used a silver grating to excite SPPs for 16-fold green UCL enhancement [36]. In these studies, the SPP resonance matched to the absorption wavelength of UCNPs. Indeed, an increase in the absorption process is anticipated to result in better UCL enhancement, especially for weak excitation [15]. It is worth noting that UCL enhancements in the literature are relative since the enhancement factor depends on the used excitation power density and the reference sample [36].

The motivation for this work is to study how plasmonic gold gratings can enhance the UCL to be applied in biosensing applications. Gold is a preferred metal in biosensing applications due to its chemically inert nature, long-term stability, and thiol-gold association [37], [38]. Gratings can be fabricated by using low-cost and scalable methods such as nanoimprinting [33] and optical disk–based techniques [39], [40]. The gold grating was designed and optimized in COMSOL Multiphysics, aiming to match the SPP resonance with the 976 nm absorption wavelength of UCNPs. The optimal structure was fabricated by using UV nanoimprinting and gold-coating on the polymer replica by thermal evaporation. The surface of the fabricated gold gratings was functionalized with commercial core–shell UCNPs conjugated with antibodies via functional groups on the hydrophilic shell. The shell had a thickness of 12 nm and acted as a spacer between UCNPs and plasmonic surfaces to prevent quenching effects [24]. The surface functionalization mimicked the structure of functional biosensing, for example, fluorophore-linked immunosorbent assays and lateral flow assays. UCNP immobilization was indirectly achieved through covalent bonds between the conjugated antibodies of UCNPs and 3,3′-dithiobis (sulfosuccinimidyl propionate) (DTSSP) cross-linkers on the surface. This stable immobilization represents a general bioassay, which can be applied for different analytes by choosing relevant antibodies. UCL measurements were performed to study the UCL enhancement mechanism. We achieved a UCL enhancement up to 65 times at low excitation power density. The main contribution to this enhancement was the increase in the absorption process. We found a slight quenching effect in the emission process with UCNPs near the gold surfaces. Simulation results were in good agreement with experimental observations.

## 2 Materials and methods

### 2.1 Theoretical background

In this work, UCNPs were composed of NaYF_4_:Yb^3+^, Er^3+^, which exhibit efficient energy transfer upconversion (ETU) [15], [41]. In ETU process, the Yb^3+^ ion acts as an activator, which absorbs 976 nm wavelength. The Er^3+^ ion is the sensitizer, which receives energy from the Yb^3+^ ion and emits upconverting photons. The absorption cross section of an UCNP is [41]

(1) $$\sigma \approx \frac{{\left|E\left(r_{0}\right)\right|}^{2}}{{\left|E_{0}\right|}^{2}},$$

where |*E*(*r*_0_)| is the local field amplitude at the location of the UCNP, and |*E*_0_| is the incident field amplitude. In the emission process, we considered UCNPs as independent emitters with a quantum yield [42]

(2) $$\eta^{\text{p}}=\frac{\gamma_{\text{rad}}^{p}/\gamma_{\text{rad}}^{0}}{\gamma_{\text{rad}}^{\text{p}}/\gamma_{\text{rad}}^{0}+\gamma_{\text{abs}}^{\text{p}}/\gamma_{\text{rad}}^{0}+\left(1-\eta^{0}\right)/\eta^{0}},$$

where *η* ^0^ is the intrinsic quantum yield of UCNPs, *γ* ^0^_rad_ is the radiative decay rate of UCNPs in free space, and *γ* ^p^_rad_ and *γ* ^p^_abs_ are the radiative decay rate and the absorption rate in the presence of the plasmonic structure, respectively. The details are described in Section 1 in the Supplementary Information.

### 2.2 Computational modeling

**Figure 1:**
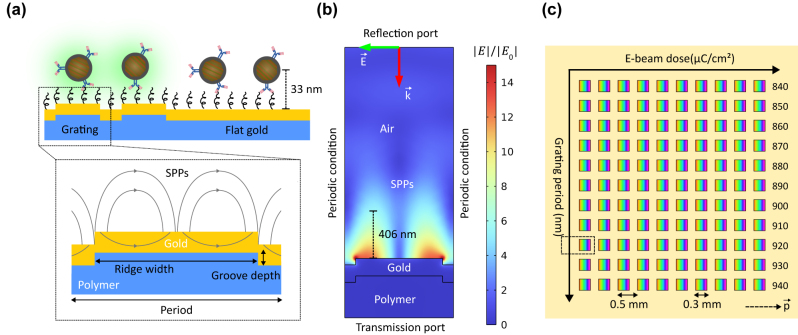
Grating-based SPP-enhanced UCL. (a) Illustration of SPP-enhanced UCL on gold grating in comparison with flat gold. (b) 2D FEM model of a grating unit cell for structural optimization with the normalized field distribution corresponding to the optimal grating. (c) The grating matrix containing 110 square-shaped gratings with an area of 0.3 × 0.3 mm^2^ each and a space of 0.2 mm between them for studying UCL enhancement as a function of the grating period. The yellow area around the gratings is flat gold. The dashed arrow shows the grating vector. The dashed rectangle indicates the scanning area including the optimal grating for enhancement factor calculation.

Plasmon-enhanced UCL aims to boost either the absorption process or emission process [18]. An increase in the absorption process results in better enhancement in UCL, especially in weak excitation [15]. Therefore, the goal is, here, to optimize the SPP resonance matching with the 976 nm absorption wavelength of UCNPs. SPPs were excited using a gold grating, as illustrated in Figure 1(a). The phase matching condition of SPP coupling on grating is [32]

(3) $$n\sin\left(\theta \right)+m\frac{\lambda }{p}=\pm \sqrt{\frac{\varepsilon_{\text{Au}}n^{2}}{\varepsilon_{\text{Au}}+n^{2}}},$$

where *n* is the refractive index of the medium above the grating surface, *ε*_Au_ is the dielectric constant of gold, *m* is the integer of the diffraction order, *θ* is the incidence angle, *λ* is the 976 nm excitation wavelength, and *p* is the grating period. Since the sample was in dry condition, we assumed *n* = 1.

#### 2.2.1 Absorption process

The structural parameters of the gold grating were optimized using the finite element method (FEM) in COMSOL Multiphysics. We approximated the 3D grating by a 2D model to reduce computational resources as shown in Figure 1(b). In the model, the polymer grating had a thickness of 300 nm and a refractive index of 1.55 (OrmoCore, Micro Resist Technology GmbH, Germany). The grating was coated with a 150 nm gold layer. The dispersion of gold was modeled by the Brendel–Bormann model from the material library in COMSOL Multiphysics. We considered a 2 µm air layer (*n* = 1) above the gold layer. Since grating is a periodic structure, we modeled it by using periodic condition along the direction of periodicity. The grating structure was illuminated from the top of the model window at a normal incidence. The illumination was a linearly transverse magnetic (TM) polarized plane wave. The reflectance and transmittance spectra were monitored from the top and the bottom of the model window, respectively. Figure 1(b) shows the normalized field distribution corresponding to the optimal grating. It had a period of 920 nm, a groove depth of 55 nm, and a duty cycle of 0.8, defined as the ratio between the ridge width and the period. To estimate the SPP decay length, we used a reference point on the grating surface and a point where the electric field was equal to 1/*e* of the reference point, as illustrated by the length marker in Figure 1(b). The decay length was 406 nm, compared to the 33-nm UCNP conjugation. Therefore, we assumed that the field decay effect was insignificant. To study the period-dependent UCL enhancement, we varied the period of the optimal grating from 840 nm to 940 nm, as shown in Figure 1(c). Furthermore, the electron-beam (E-beam) dose was gradually increased for each period to study the effect of fabrication tolerances on the grating performance. The E-beam dose for each grating is shown in Table S1. In total, the grating matrix contained 110 square-shaped gratings with an area of 0.3 × 0.3 mm^2^ each and a space of 0.2 mm between them.

#### 2.2.2 Emission process

To study the quantum yield of UCNPs near plasmonic surfaces, we modeled the decay rates in the green emission process of UCNPs. We assumed that each UCNP was infinitely small and emitted light in all directions randomly. In COMSOL Multiphysics, we used an electric dipole to model the UCNP. The electric dipole oscillated in all directions at the frequency corresponding to the wavelength of 540 nm in vacuum. The decay rates were monitored to calculate the quantum yield in the presence of plasmonic surface. The details are described in Section 3 in the Supplementary Information.

### 2.3 Fabrication

The grating matrix was written by using E-beam lithography and subsequently transferred to a silicon master by reactive ions etching. The silicon master was then replicated to a polymeric material by using UV nanoimprinting. Finally, the replicates were coated with a 5 nm chromium layer as an adhesive layer and then a 150 nm gold layer. The details of the fabrication process are described in our previous work [43]. Scanning electron microscope (SEM) images of the silicon master and gold-coated replicates are shown in Figure S2.

### 2.4 UCNP immobilization

**Figure 2:**
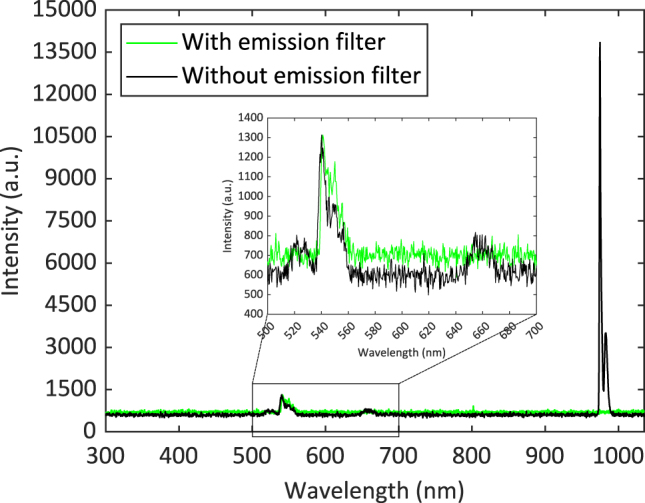
Emission spectra of the used UCNP-CRP Abs with and without an emission filter (540 nm–580 nm) in the experimental setup under 976 nm excitation.

UCNPs were conjugated with antibodies and indirectly immobilized on the surface of the grating matrix using 3,3′-dithiobis (sulfosuccinimidyl propionate) (DTSSP) cross-linking, as illustrated in Figure 1(a). UCNPs (UPCON 540, product no. 43-07, 8.8 mg/ml UCNP, Kaivogen Oy) were conjugated with antibodies (C-reactive protein antibody, Anti-h CRP 6405 SPTN-5, Medix Biochemica) as a custom service by Kaivogen Oy. The core of UCNPs had a diameter of 36 nm and was composed of NaYF_4_:Yb^3+^, Er^3+^. UCNPs had a 12 nm hydrophilic shell. C-reactive protein antibody (CRP Ab) was conjugated with the hydrophilic shell to form UCNP-CRP Ab. The emission spectrum of UCNP-CRP Ab is shown in Figure 2.

The surface of the grating matrix was functionalized with a water-soluble sulfonated form of Lomant’s reagent [44], DTSSP (Sigma-Aldrich, 322133), to add amine-reactive sites on the surface [45]. DTSSP linkers adsorbed onto the surface through the disulfide group [46], and its terminal succinimidyl groups [45] allow covalent attachments of amino-containing biomolecules [45], [47]. UCNP-CRP Abs were attached to the self-assembled DTSSP monolayer by covalent bonds formed between the CRP Ab and the active sulfosuccinimide easter groups of DTSSP. TSA buffer (50 mM Tris-HCl, 0.9 % NaCl, 0.05 % NaN_3_, 0.01 % Tween20, 0.5 % BSA & 1 mM KF in dH_2_O) was used as washing buffer after the immobilization of UNCP-CRP Ab. To stabilize the UNCP-CRP Ab during long incubations and washes, UCNP stabilizer (50× concentrate, product no. 46-19, Kaivogen Oy) was supplied in the buffer solutions. The detailed protocol is described in Section 5 in the Supplementary Information.

### 2.5 Experimental setup

**Figure 3:**
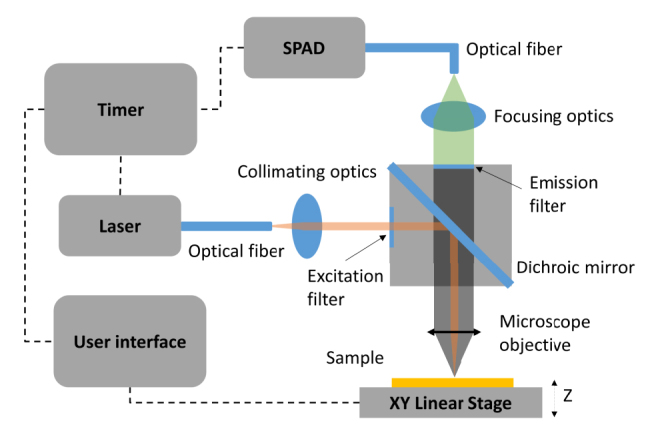
Schematic of the experiment setup for UCL intensity raster scanning using an *XY* linear stage. The laser operates in continuous-wave mode for scanning UCL measurements and in pulsed mode for time-resolved measurements.

The experimental setup is illustrated in Figure 3. The excitation source was a picosecond laser (LDH-D-F-980, PicoQuant) in continuous-wave operation mode with a central wavelength of 976 nm and a spectral bandwidth of 1 nm. For time-resolved measurements, the laser worked at pulsed mode with a spectral bandwidth of 15 nm. A single photon avalanche detector (SPAD) (SPD_VIS_M1, Aurea Technology) was synchronized to the laser by an event timer. A high-speed motorized *XY* linear stage (MLS203-1, Thorlabs) was used to perform UCL intensity raster scanning. The repeatability and the minimum incremental movement of the stage was 0.25 µm and 0.1 µm, respectively. The excitation beam from the laser was guided by a polarization maintaining single mode fiber (PM980-XP, Thorlabs). The beam was collimated (F810APC-850, Thorlabs) and then filtered by an excitation filter (ET780lp, Chroma Technology) in one side of the filter cube to prevent any side modes of the laser. The collimated beam was reflected by a dichroic mirror (DMSP805R, Thorlabs) and focused on the sample by a 10× objective (N10X-PF-10X, Nikon) to excite UCNPs on the surface of the grating matrix. The diameter of the collimated beam in front of the objective was estimated to be around 7.8 mm. The scattering light and upconversion emissions were collected by the same objective. They were filtered by an emission filter with a band-pass range from 540 nm to 580 nm (ET560/40×, Chroma Technology). The filtered light was coupled by a collimator (F810FC-543, Thorlabs) to a multimode fiber (FG050LGA, Thorlabs) and guided to SPAD.

## 3 Results and discussion

### 3.1 Absorption enhancement

#### 3.1.1 Surface plasmon polariton excitation

**Figure 4:**
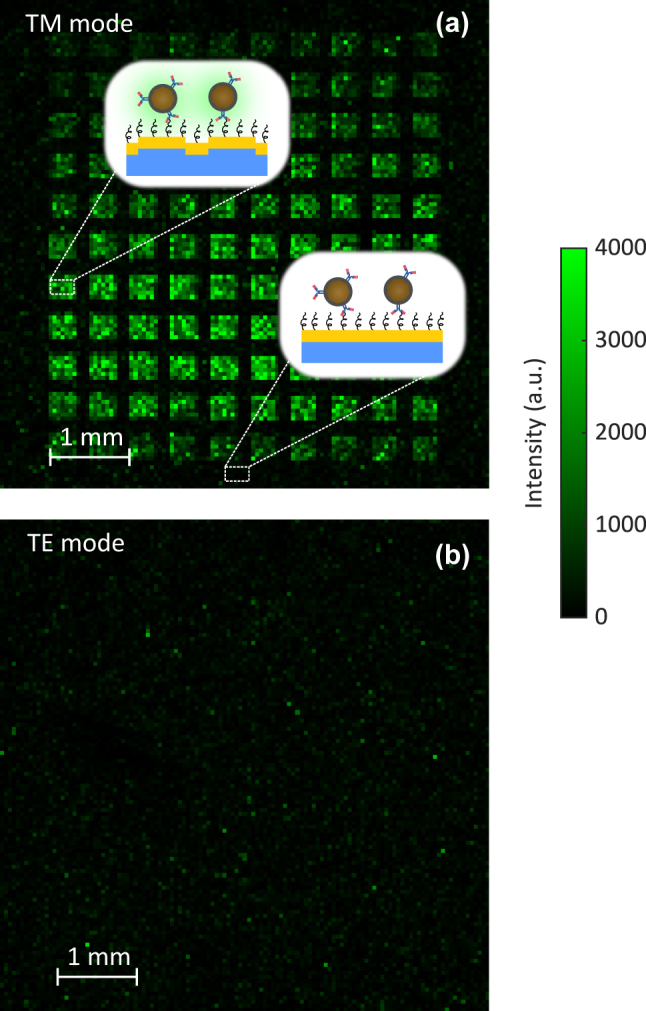
UCL measurement of the grating matrix with (a) TM polarization and (b) TE polarization. The scanning area is 6 × 6 mm^2^ with an incremental *XY* linear stage movement of 50 µm. The insets illustrate functionalized UCNPs on the surface of the matrix.

The grating matrix with functionalized UCNPs was characterized with the experimental setup. Figure 4(a) shows the obtained UCL intensity of the matrix under TM illumination. The scanning area was 6 × 6 mm^2^ with an incremental *XY* linear stage movement of 50 µm. When rotating the grating, the intensity gradually decreased, reaching a minimum at TE configuration. The corresponding TE illumination result in Figure 4(b) was obtained by rotating the matrix with an angle of 90°. In Figure 4, the matrix was clearly visible under TM illumination, while it was invisible with TE polarization. This was expected since SPPs are only excited with TM polarization. The excitation of SPPs on the grating surface led to an increase in the local field at the location of UCNPs. Therefore, the absorption cross section of UCNPs was enhanced, resulting in higher UCL intensity in comparison with the flat gold surrounding the grating matrix or gratings under TE illumination.

#### 3.1.2 Focused Gaussian beam analysis

**Figure 5:**
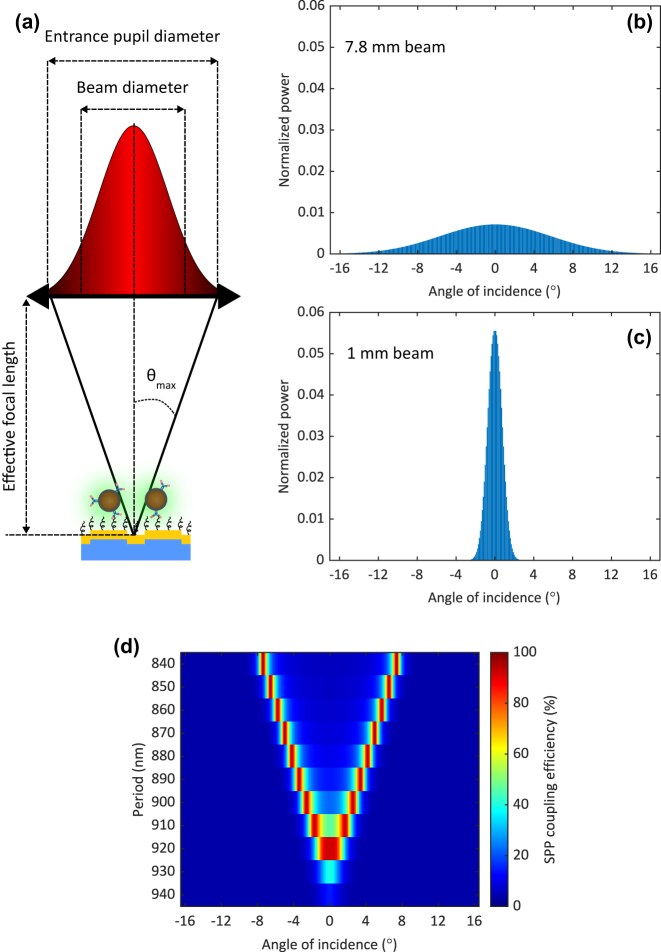
Focused Gaussian beam analysis. (a) Illustration of focused Gaussian beam on the grating surface. The maximum obtainable incidence angle is limited by the entrance pupil diameter and the effective focal length of the objective. Normalized power distribution as a function of incidence angle of (b) 7.8 mm beam and (b) 1 mm beam. (d) Computational SPP coupling efficiency as a function of incidence angle of gratings with a groove depth of 55 nm, a duty cycle of 0.8, and a varying period from 840 nm to 940 nm.

The UCL intensity depended on the grating period, excitation wavelength, and incidence angle. With the excitation wavelength fixed, the intensity varied with the grating period and the incidence angle. We optimized the grating with a period of 920 nm for the normal-incidence Gaussian beam used in the experimental setup in Figure 3. Tilted Gaussian beams would result in decrease in the intensity. A normal-incidence Gaussian beam is illustrated in Figure 5(a). The maximum incidence angle was limited by the entrance pupil diameter and the effective focal length of the objective. In our setup, the maximum obtainable incidence angle was 16.5°. Gaussian beams with different diameters exhibit varying SPP coupling efficiencies for each period in the grating matrix. To investigate this, we compared UCL intensities computationally and experimentally of two sets of collimators (F810APC-850 and CFP5-980A, Thorlabs), namely, 7.8 mm beam and 1 mm beam.

In COMSOL Multiphysics, the focused Gaussian beam was modeled by decomposing the beam into plane waves with an incidence angle varying from −16.5° to +16.5° and a sampling step of 0.1°. The normalized power distributions as a function of incidence angle, *P*(*θ*), for 7.8 mm and 1 mm beams are shown in Figure 5(b) and (c). The details are described in Section 6 in the Supplementary Information. We modeled the SPP coupling efficiency as a function of incidence angle *η*(*θ*), shown in Figure 5(d), for gratings with a groove depth of 55 nm, a duty cycle of 0.8, and a varying period from 840 nm to 940 nm, corresponding to the period of the grating matrix in Figure 1(c). For each period, the computational UCL intensity was

(4) $$I_{\text{si}}\approx \overset{+16.5^{◦}}{\underset{\theta =-16.5^{◦}}{\sum }}P\left(\theta \right)\times \eta \left(\theta \right).$$

**Figure 6:**
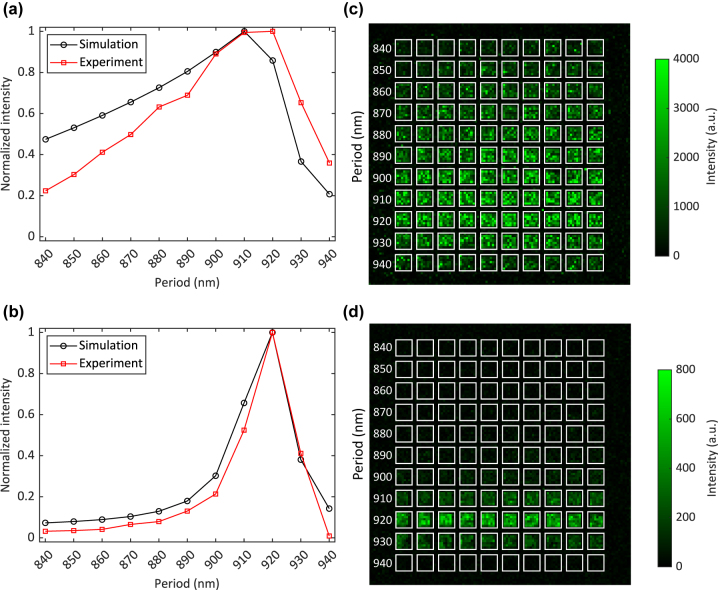
Effect of focused Gaussian beams on SPP-enhanced UCL. Comparison between simulation and experiment of normalized UCL intensity as a function of grating period when using (a) a 7.8 mm beam and (b) a 1 mm beam. Experimental UCL intensities with beam diameters of (c) 7.8 mm and (d) 1 mm in front of the objective in the experimental setup. The white squares indicate the gratings in the matrix.

The computational UCL intensity was then normalized with the maximum intensity and plotted in Figure 6(a) and (b). Computationally, we found that when narrowing the beam diameter, the intensity exhibited the highest value at the optimal period of 920 nm and dropped significantly at other periods. This was the result of compressing the excitation power closer to the normal incidence angle as shown in Figure 5(c).

Experimentally, we replaced the 7.8 mm beam with the 1 mm beam and measured the UCL intensity of the grating matrix at the same laser power and incremental movement. The measured UCL intensities of the grating matrix caused by the 7.8 mm beam and the 1 mm beam are shown in Figure 6(c) and (d), respectively. It is worth noting that the focal spot diameter was different between the 7.8 mm beam and the 1 mm beam (3.2 µm and 24.9 µm, respectively) at the excitation wavelength of 976 nm. The UCL intensity dropped as the result of the decrease in power density, i.e., 4.35 kW/cm^2^ and 0.07 kW/cm^2^, respectively. As predicted by the simulations, the 920-nm period gratings in the matrix in Figure 6(d) exhibit the highest UCL intensity with the 1 mm beam. To compare with simulation results, the measured UCL intensity was area-averaged for each individual grating in the matrix. For each period, the experimental UCL intensity was

(5) $$I_{\text{ex}}=\frac{\overset{10}{\underset{k=1}{\sum }}{\bar{I}}_{k}}{10}.$$

The experimental UCL intensity was then normalized with the maximum intensity and plotted in Figure 6(a) and (b). There was a good agreement between the simulation and experiment. We also found that fabrication tolerances led to variations in the measured UCL intensity within the 10 nominally similar gratings, as shown in Figure S3. Another factor contributing to these variations was the nonuniform distribution of UCNPs, which can be observed in Figure 4(b). In the following sections, we used the 1 mm beam to investigate the quenching effect and UCL enhancement.

### 3.2 Quenching effect in the emission process

In Section 3.1, we found that the increase in the local field, caused by the SPP coupling on the grating surface, led to UCL enhancement. However, the presence of the plasmonic surface can cause also a strong quenching effect in the emission process [19], [24]. Feng et al. and Greybush et al. showed that there was a strong quenching effect when the distance between UCNPs and plasmonic surfaces was below 8 nm [21], [24]. In this work, core–shell UCNPs with a shell thickness of 12 nm were used to minimize the quenching effect. Computationally and experimentally, we investigated the quenching effect on flat gold and grating. Supported by the SEM images of the grating matrix with UCNP immobilization in Figure S4, we made the assumption that, since UCNPs were distributed individually on the surface, only a single UCNP would be present within the focal spot.

**Figure 7:**
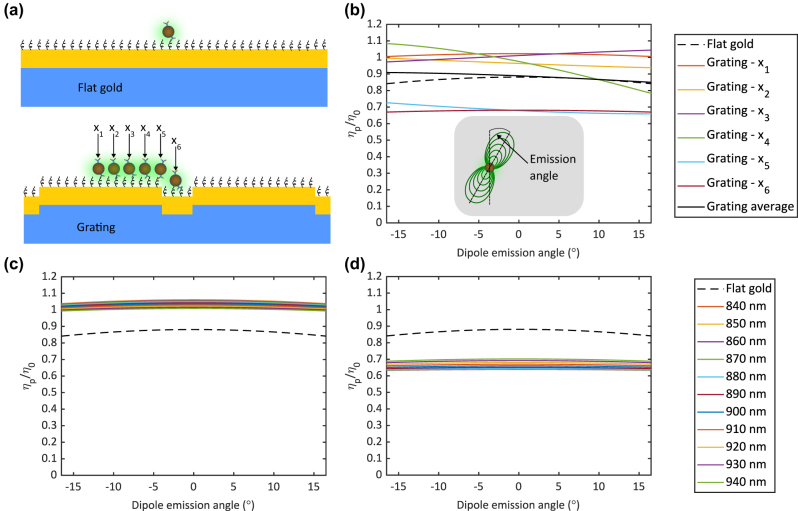
Computational investigation of the quenching effect with a UCNP near a gold surface. (a) Illustration of the location of the UCNP on flat gold or on grating with six cases (*x*_1_, *x*_2_, *x*_3_, *x*_4_, *x*_5_, and *x*_6_). (b) The quenching effect of the UCNP on flat gold and on grating as a function of the dipole emission angle. The quenching effect is evaluated by the quantum yield ratio of the UCNP near the gold surface (*η*_p_) and in free space (*η*_0_). The dipole emission angle is determined by the collection angle of the used objective. The quenching effect with the UCNP at the locations (c) *x*_1_ – on the ridge and (d) *x*_6_ – inside the groove of gratings with a period from 840 nm to 940 nm.

In Figure 7(a), we computationally studied the quenching effect when a UCNP was on flat gold or on grating at six cases *x*_1_, *x*_2_, *x*_3_, *x*_4_, *x*_5_, and *x*_6_. Case *x*_1_ featured a UCNP positioned in the middle of the grating ridge, whereas case *x*_6_ corresponded to a UCNP placed in the middle of the grating groove. The spacing between two consecutive cases was defined as (0.5 × period)/5. The quantum yield ratio was calculated as a function of the dipole emission angle when the UCNP was either near the gold surface (*η*_p_) or in the free space (*η*_0_). The quenching effect happened when this ratio was smaller than 1. The dipole emission angle was from −16.5° to +16.5°, corresponding to the collection angle of the used 10× objective with a numerical aperture of 0.3. As shown in Figure 7(b), flat gold caused a slight quenching effect. On grating, the quenching effect depended on the location of the UCNP. When the UCNP was on the ridge (*x*_1_, *x*_2_, and *x*_3_), there was no quenching effect. However, when the UCNP was near or inside the groove (*x*_4_, *x*_5_, and *x*_6_), the groove acted as a light-trapping cavity, causing a strong quenching effect. The curves in cases *x*_1_ and *x*_6_ exhibited a symmetry similar to that of flat gold. Cases *x*_2_, *x*_3_, *x*_4_, and *x*_5_ showed asymmetric curves, depending on the distance between the UCNP and the groove. By taking an average of six cases of the UCNP on grating (solid black line), the quenching effect was similar to flat gold (dashed black line). In Figure 7(c) and (d), we investigate the influence of the grating period on the quenching effect at the locations *x*_1_ and *x*_6_, respectively. The quenching variations among the periods were negligible.

**Figure 8:**
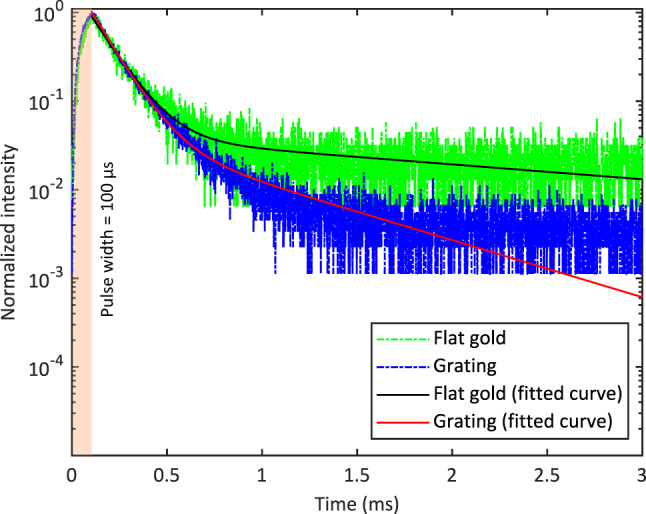
Time-resolved UCL measurements on flat gold and on one of the 920 nm gratings and their corresponding fitted exponential decay curves. The excitation pulse width is 100 µs.

Experimentally, we conducted time-resolved UCL measurements on flat gold and on one of the 920 nm gratings. To improve the signal-to-noise ratio, particularly for the time-resolved UCL measurement on the flat gold, a 100× objective was used. The measurement results are presented in Figure 8. The excitation pulse width was 100 µs. The data were smoothened with the Savitzky–Golay filter in MATLAB and normalized in the range from 0 to 1. To determine the decay rate, the experimental decay data were fitted with exponential curves, as shown in Figure 8. Both the flat gold and the grating exhibited rather similar radiative decay times, which were 138 µs and 125 µs, respectively. Therefore, the quenching effect was comparable between the grating and the flat gold, aligning with the expectations from the computational investigations. Furthermore, this was in agreement with the observation in Figure 4(b). When there was no absorption enhancement under TE illumination, the difference in UCL intensity between the gratings and flat gold was not observable.

### 3.3 Upconversion luminescence enhancement

**Figure 9:**
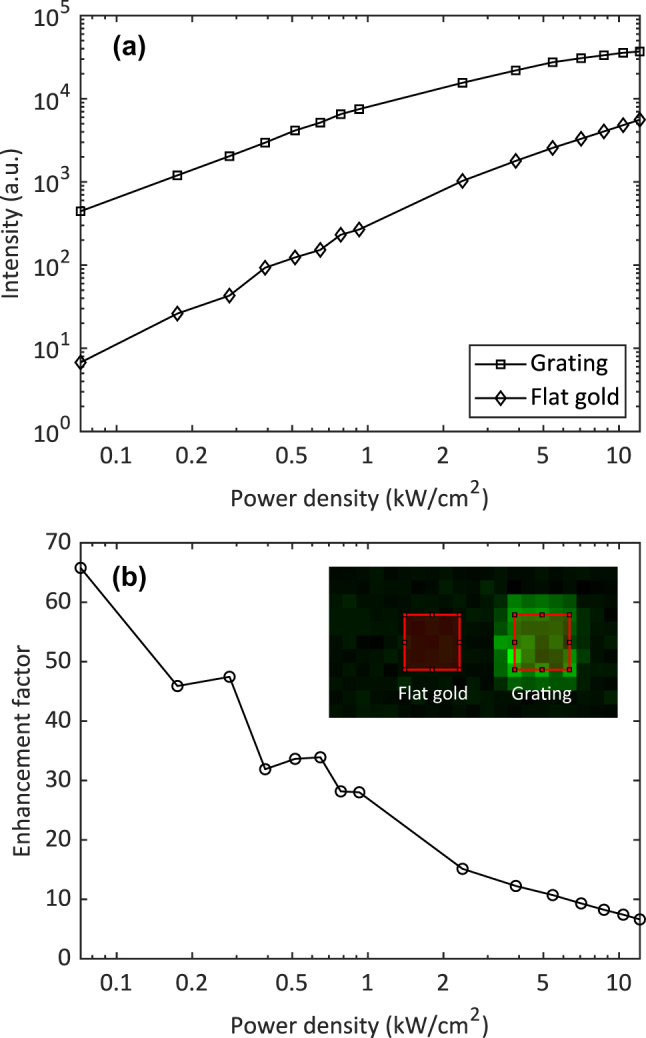
Experimental UCL enhancement. (a) Area-averaged UCL intensities on grating and on flat gold as a function of excitation power density. The area-averaged UCL intensities are subtracted with the SPAD background noise (around 50 photons). (b) Enhancement factor as a function of excitation power density. The enhancement factor is the ratio between averaged UCL intensity on grating and on flat gold.

The UCL enhancement was calculated by taking the ratio of the area-averaged UCL intensity between grating and flat gold. We scanned a 0.5 × 1.0 mm^2^ area around a grating, as illustrated in the dashed rectangle in Figure 1(c), using various laser powers. The raster scan was done with an incremental *XY* linear stage movement of 50 µm. The UCL measurement is shown in the inset in Figure 9(b). We masked the grating and the flat gold with the red squares having an area of 0.2 × 0.2 mm^2^. The masking area was smaller than the grating area (0.3 × 0.3 mm^2^) to avoid the edge effect. UCL intensities were area-averaged for the grating and the flat gold. The averaged UCL intensities was then subtracted from the SPAD background noise (around 50 photons) and plotted as a function of power density in Figure 9(a). The enhancement factor is shown in Figure 9(b). At low power density, it exceeded 40 and gradually decreased to below 10 as the power density increased. This was consistent with the theory of the UCL enhancement in absorption process [15]. In particular, the enhancement factor is proportional to |*E*/*E*_0_|^4^ at low power density and |*E*/*E*_0_|^2^ at high power density for the two-photon upconversion process [15]. This enhancement factor is comparable to those reported in the literature. For instance, a 17-fold enhancement has been reported at a power density of 1 kW/cm^2^ [36], compared to our 28-fold enhancement at the same power density. At a very high power density of 100 kW/cm^2^, enhancement factors of 2.4-fold [35] and 4-fold [36] have been reported, while we achieved an 8-fold enhancement at the maximum power density of 10 kW/cm^2^. However, it is worth noting that there was a slight quenching effect when UCNPs were on flat gold and grating, which might affect the enhancement factor. We also evaluated the UCL enhancement using a commercial UCL reader (Labrox Oy, Turku, Finland). The results are discussed in Section 10 in the Supplementary Information.

## 4 Conclusions

In summary, we demonstrated SPP-enhanced UCL using a gold grating. The gold grating was optimized in COMSOL Multiphysics to match the SPP resonance with the 976 nm absorption wavelength of UCNPs at normal incidence. The optimal grating had a groove depth of 55 nm, a duty cycle of 0.8, and a period of 920 nm. To investigate the UCL enhancement mechanism, we fabricated a grating matrix with the optimal groove depth and duty cycle, while varying the periods from 840 nm to 940 nm. The matrix was then functionalized with UCNPs. SPP coupling on the gratings in the matrix led to an increase in the local field of UCNPs. Therefore, the absorption cross section of UCNPs was enhanced, resulting in strong UCL emission intensities on the gratings. We also investigated the focusing effect of the Gaussian beams on the SPP-enhanced UCL on the gratings in the matrix. When narrowing the beam diameter in front of the objective, the highest UCL intensity was observed on the gratings with the optimal period of 920 nm, whereas it dropped significantly at other periods. This was due to the excitation power compression closer to the normal incidence angle. Furthermore, the quenching effect caused by a gold surface was computationally studied. We found that there was a slight quenching effect with UCNPs near the gold surface and negligible difference between the flat gold and grating. The experimental observations in time-resolved measurements and UCL measurements under TE illumination supported these computational results. Finally, we calculated the UCL enhancement factor using the flat gold as a reference. We achieved an enhancement factor up to 65 at low excitation power density, i.e., 0.07 kW/cm^2^. Indeed, low excitation power density is preferred in UCL imaging systems. For instance, to image a 1 × 1 mm^2^ area with a power density of 0.07 kW/cm^2^, the grating requires a laser power of 0.7 W, whereas for the flat gold to achieve comparable UCL intensity, as estimated 15 W of laser power is needed. Furthermore, the enhancement in UCL will improve the signal-to-noise ratio, thereby enhancing the sensitivity of UCNP-based biosensors.

## Supplementary Information

See the Supplementary Information for additional information: quantum yield in emission process; electron beam dose; decay rate simulation; scanning electron microscopy images; UCNP immobilization; focused Gaussian beam analysis; focal spot size; UCL intensity variation; UCNP distribution on the surface; SPP-enhanced UCL measurement with a commercial reader.

## Supplementary Material

Supplementary Material Details
