# Supplementary material for: Surface plasmon polariton–enhanced upconversion luminescence for biosensing applications
Source: Nanophotonics. 2024 Aug 27;13(21):3995–4006. doi: 10.1515/nanoph-2024-0247 (PMC11501061; doi:10.1515/nanoph-2024-0247)
Supplement: Supplementary file 1 — Supplementary Material Details [file j_nanoph-2024-0247_suppl_001.pdf]

Supplementary Information

Duc Le*, Marjut Kreivi, Sanna Aikio, Noora Heinilehto, Teemu Sipola, Jarno Petäjä, Tian-Long Guo, Matthieu Roussey, and Jussi Hiltunen

Surface plasmon polariton enhanced upconversion luminescence for biosensing **applications**

1. Quantum yield in emission process

In emission process, we consider UCNP as a light emitter with the intrinsic quantum yield[1]

$$\eta^0 = \frac{\gamma_{\mathrm{rad}}^0}{\gamma_{\mathrm{rad}}^0 + \gamma_{\mathrm{nonrad}}^0}, \tag{1}$$

where γ_{rad}^0 and $\gamma_{\mathrm{nonrad}}^0$ are the radiative and nonradiative decay rates, respectively. When the UCNP is near plasmonic surface, the quantum yield is[1]

$$\eta^{\mathrm{p}} = \frac{\gamma_{\mathrm{rad}}^{\mathrm{p}}}{\gamma_{\mathrm{rad}}^{\mathrm{p}} + \gamma_{\mathrm{nonrad}}^{\mathrm{p}} + \gamma_{\mathrm{abs}}^{\mathrm{p}}}, \tag{2}$$

where $\gamma_{\mathrm{rad}}^{\mathrm{p}}$, $\gamma_{\mathrm{nonrad}}^{\mathrm{p}}$, and $\gamma_{\mathrm{abs}}^{\mathrm{p}}$ are the radiative decay rate, nonradiative decay rate, and absorption rate in the presence of plasmonic structure, respectively. We assumed the nonradiative decay rate of the UCNP to be independent of the plasmonic structure, i.e., $\gamma_{\mathrm{nonrad}}^0 = \gamma_{\mathrm{nonrad}}^{\mathrm{p}}$. Insert (1) to (2), we have

$$\eta^{\mathrm{p}} = \frac{\gamma_{\mathrm{rad}}^{\mathrm{p}}/\gamma_{\mathrm{rad}}^0}{\gamma_{\mathrm{rad}}^{\mathrm{p}}/\gamma_{\mathrm{rad}}^0 + \gamma_{\mathrm{abs}}^{\mathrm{p}}/\gamma_{\mathrm{rad}}^0 + (1-\eta^0)/\eta^0}. \tag{3}$$

2. Electron beam dose

Table S1. The electron beam dose applied for each grating in the matrix during E-beam lithography process.

Period (nm)	E-beam dose (μC/cm^2)									
840	105.00	104.75	104.50	104.25	104.00	103.75	103.50	103.25	103.00	102.75
850	102.50	102.25	102.00	101.75	101.50	101.25	101.00	100.75	100.50	100.25
860	100.00	99.75	99.50	99.25	99.00	98.75	98.50	98.25	98.00	97.75
870	97.50	97.25	97.00	96.75	96.50	96.25	96.00	95.75	95.50	95.25
880	95.00	94.75	94.50	94.25	94.00	93.75	93.50	93.25	93.00	92.75
890	92.50	92.25	92.00	91.75	91.50	91.25	91.00	90.75	90.50	90.25
900	90.00	89.75	89.50	89.25	89.00	88.75	88.50	88.25	88.00	87.75
910	87.50	87.25	87.00	86.75	86.50	86.25	86.00	85.75	85.50	85.25
920	85.00	84.75	84.50	84.25	84.00	83.75	83.50	83.25	83.00	82.75
930	82.50	82.25	82.00	81.75	81.50	81.25	81.00	80.75	80.50	80.25
940	80.00	79.75	79.50	79.25	79.00	78.75	78.50	78.25	78.00	77.75

3. Decay rate **simulation**

The quantum yield of an UCNP near a plasmonic surface was calculated based on equation (3). The intrinsic quantum yield η^0 of the UCNP was 0.03[2]. The decay rates were modeled using an electric dipole oscillating at the wavelength of 540 nm in air (*n* = 1), as illustrated in Figure S1. We assumed that UCNPs emitted photons in all directions randomly. However, the used objective had a numerical aperture of 0.3 with a calculated maximum angle of collection of 16.5°. Therefore, we modeled the electric dipole emitting light at the angle from -16.5° to +16.5°. The 2D model window dimension was 7360 nm × 7360 nm (8 × grating period), which was large enough in comparison with the SPP propagation length of 540 nm on the gold surface, i.e., 946 nm. A perfectly matched layer surrounded the model window with a thickness of 540 nm. The dipole was placed above the gold surface at 33 nm, considering the DTSSP-antibody conjugation, the hydrophilic layer, and the radius of the UCNP. We estimated the length of DTSSP-antibody conjugation to be around 3 nm in its dry stage. The hydrophilic layer had a thickness of 12 nm, and the radius of the UCNP was 18 nm. The radiative power was calculated by integrating the power along the edge of the far-field window marked by the red square in Figure S1

$$P_{\mathrm{rad}} = \int_l (\mathrm{ewfd.nPoav})\mathrm{d}l, \tag{4}$$

where $\mathrm{ewfd.nPoav}$ is the time-averaged power in COMSOL Multiphysics. The absorbed power was the integration of ohmic-loss rate over the area of gold, which is

$$P_{\mathrm{abs}} = \int_A (\mathrm{ewfd.Qrh})\mathrm{d}A, \tag{5}$$

where $\mathrm{ewfd.Qrh}$ is the ohmic-loss rate in COMSOL Multiphysics. The Poynting's theorem gives the relation between the power and the decay rates as[1]

$$\gamma_{\mathrm{rad}}^{0} \propto \frac{P_{\mathrm{rad}}^{0}}{2|p_{\mathrm{em}}|^2}, \gamma_{\mathrm{rad}}^{\mathrm{p}} \propto \frac{P_{\mathrm{rad}}^{\mathrm{p}}}{2|p_{\mathrm{em}}|^2}, \gamma_{\mathrm{abs}}^{\mathrm{p}} \propto \frac{P_{\mathrm{abs}}^{\mathrm{p}}}{2|p_{\mathrm{em}}|^2} \tag{6}$$

where p_{em} is the dipole moment, P_{rad}^{0} is the radiative power of the dipole in free space in Figure S1(c), $P_{\mathrm{rad}}^{\mathrm{p}}$ is the radiative power of the dipole near the plasmonic surface in Figure S1(a) and (b), and $P_{\mathrm{abs}}^{\mathrm{p}}$ is the absorbed power by the plasmonic structure in Figure S1(a) and (b). It is worth noting that the meshing element must be similar in the three models in Figure S1 since the integration depends on the meshing resolution. Therefore, we kept the same meshing element for the three models and changed the material properties for each. Insert equations (6) into equation (3), we have

$$\eta^{\mathrm{p}} = \frac{P_{\mathrm{rad}}^{\mathrm{p}}/P_{\mathrm{rad}}^{0}}{P_{\mathrm{rad}}^{\mathrm{p}}/P_{\mathrm{rad}}^{0} + P_{\mathrm{abs}}^{\mathrm{p}}/P_{\mathrm{rad}}^{0} + (1-\eta^0)/\eta^0}. \tag{7}$$

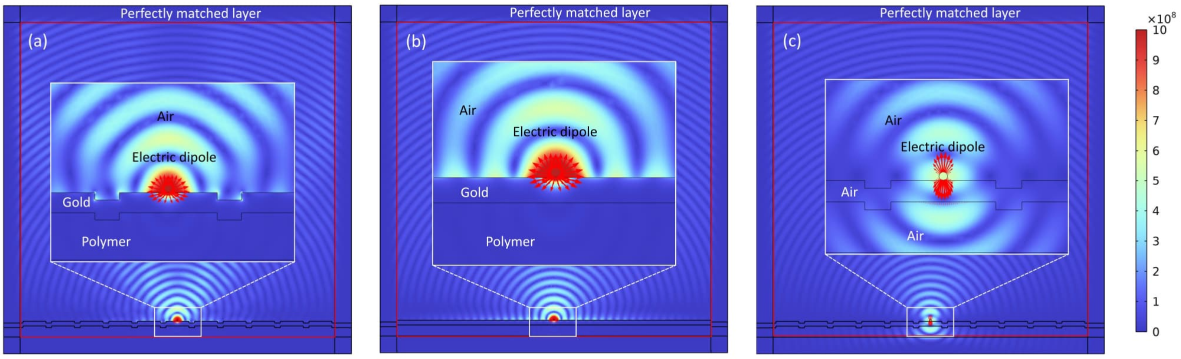

Figure S1: Decay rate simulation by using an electric dipole for calculating the quantum yield of UNCP in the presence of a gold surface. (a) The dipole near the grating surface. (b) The dipole near the flat gold surface. (c) The dipole in free space. The meshing element is identical for the three models.

4. Scanning electron microscopy images

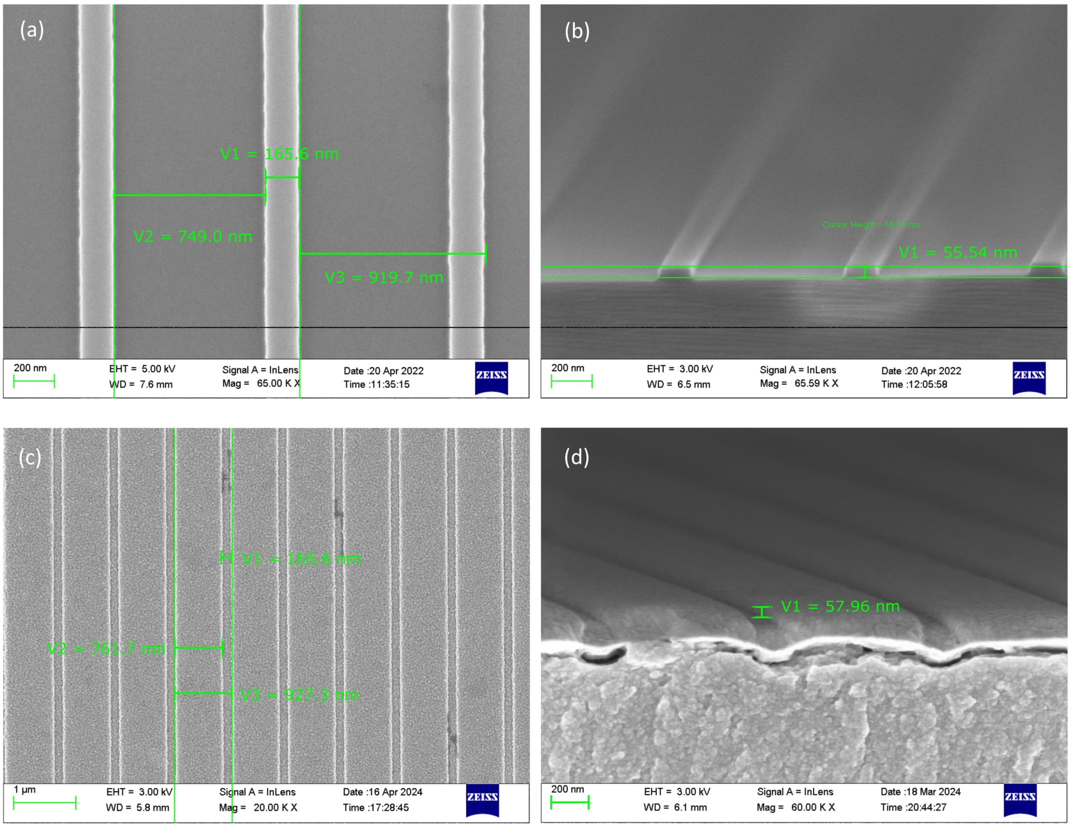

Figure S2. SEM images of the silicon master and the gold-coated replicate. (a) Top-view and (b) side-view of the silicon master. (c) Top-view and (d) side-view of the gold-coated replicate.

5. UCNP **immobilization**

DTSSP functionalization. A 4 mM DTSSP solution was prepared in 5 mM sodium citrate buffer with a pH of 5. The surface of the grating matrix was cleaned by rinsing with acetone, then milli-Q water, and eventually ethanol. The surface was then dried with a nitrogen gun and treated with O_2 plasma at 200 W for 1 minute (Technics Plasma GmbH Tepla, 440-G). The cleaned surface was immersed in the DTSSP solution and placed in an orbital shaker (Heidolph Unimax, 1010) at 60 rpm for 6 hours. The surface was then washed with milli-

Q water for 5 seconds and dried with a nitrogen gun. The DTSSP-functionalized surface was stored under a nitrogen atmosphere.

UCNP immobilization. UCNP-CRP Ab solution was diluted at a concentration of 17,6 µg/ml in phosphate-buffered saline (PBS) supplied with UCNP stabilizer. For the immobilization incubation a well plate was blocked with 1% bovine serum albumin in PBS and placed in an orbital shaker (Heidolph Unimax, 1010) at 100 rpm for 1 hour. The well plate was washed with PBS three times 5 minutes, and then rinsed twice with milli-Q water. The DTSSP-functionalized surface was immersed and incubated in the well plate with the UCNP-CRP Ab solution in an orbital shaker (Heidolph Unimax, 1010) at 100 rpm for 1 hour. The surface was then washed with TSA buffer to remove unbound UCNP-CRP Abs. The surface was shortly dipped in a tube of TSA buffer, then moved to another tube with TSA buffer supplied with UCNP stabilizer. The surface was placed vertically for gravitational wash at +4°C overnight. The surface was finally washed with milli-Q water and dried with a nitrogen gun.

6. Focused Gaussian beam analysis

The 10× objective in our experimental setup had an effective focal length (EFL) of 20 mm and an entrance pupil diameter of 12 mm. The maximum incidence angle was limited by the entrance pupil and calculated as

$$\theta_{\max} = \operatorname{atan}\frac{0.5 \times \text{entrance pupil diameter}}{\text{effective focal length}}, \tag{8}$$

which was 16.5°. Since the beam was with a Gaussian profile, the intensity distribution is defined as

$$I(r,z) = I_0\left(\frac{w_0}{w(z)}\right)^2 \exp\left(\frac{-2r^2}{w(z)^2}\right), \tag{9}$$

where r is the radial distance from the center axis of the beam and w_0 is the waist radius at $z = 0$. The intensity distribution of the collimated beam in front of the objective can be approximated

$$I(r) \approx \exp\left(\frac{-2r^2}{w_0^2}\right). \tag{10}$$

The power of a Gaussian beam is

$$P_0 = \int_{-\infty}^{+\infty} I(r)\mathrm{d}r. \tag{11}$$

The normalized power as a function of incidence angle with 0.1° sampling step is

$$P(\theta) = \frac{\int_{EFL\cdot\tan(\theta)}^{EFL\cdot\tan(\theta+0.1°)} I(r)\mathrm{d}r}{P_0}. \tag{12}$$

7. Focal spot size

The diameter of the focused Gaussian spot is

$$2w_0 = \frac{4 \times M^2 \times \lambda \times EFL}{\pi \times D}, \tag{13}$$

where λ is the excitation wavelength (976 nm), EFL is the effective focal length (20 mm), D is the beam diameter in the front of the objective (7.8 mm and 1 mm), and M^2 is the beam quality (M^2 = 1). The calculated diameter was 3.2 µm and 24.9 µm, respectively.

8. **UCL intensity variation**

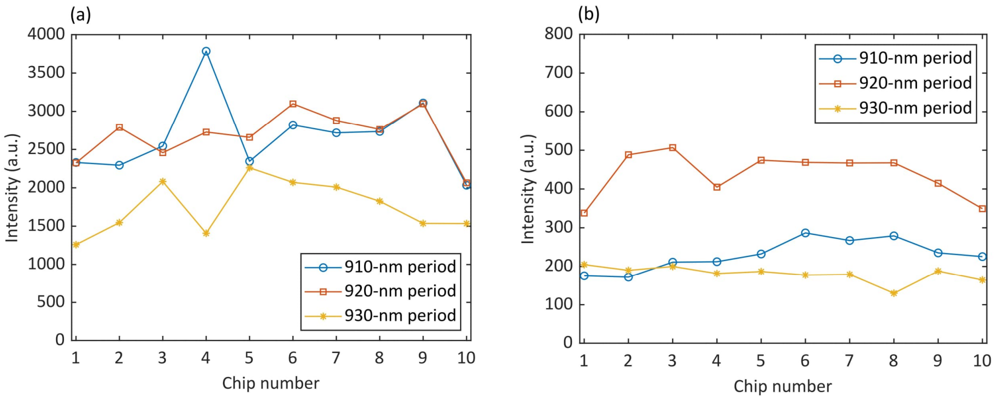

Figure S3: UCL intensity variation within 10 gratings of the periods 910 nm, 920 nm, and 930 nm when using (a) a 7.8-mm beam and (b) a 1-mm beam.

9. **UCNP distribution** on the surface

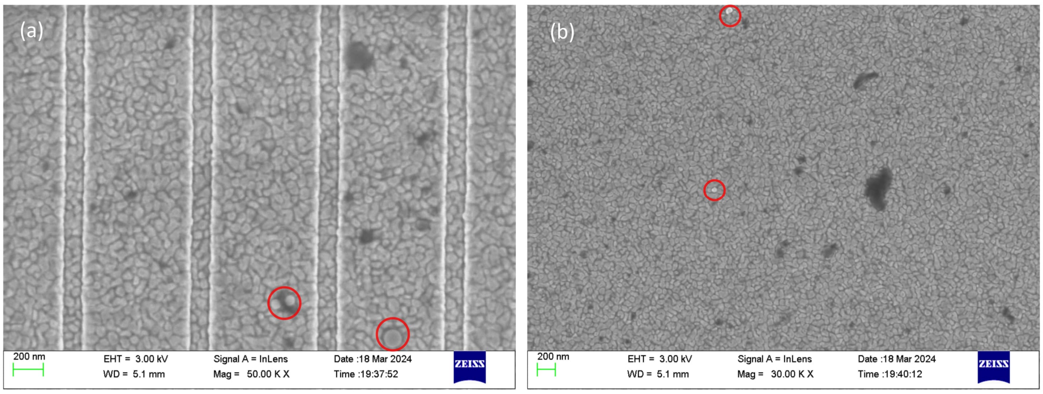

Figure S4: Top-view SEM images of the grating matrix with UCNP immobilization: (a) grating region and (b) flat gold region. The red circles indicate the location of UCNPs.

10. SPP-enhanced UCL measurement with a commercial reader

SPP-enhanced UCL was demonstrated with a commercial UCL reader (Labrox Oy, Turku, Finland). UCL measurements of this reader is based on point-to-point scanning, which is similar to our experimental setup. The excitation and emission filters were 976 nm and 540 nm, respectively. The excitation power was 10 mW. When using commercial reader, we were not able to estimate the focal spot size on the surface to calculate the excitation power density. The exposure time was 300 ms. The scanning step was 0.2 mm, which was comparably large with the grating size, i.e., 0.3 mm × 0.3 mm. As a result, the grating matrix was blurry in Figure S5(a). The background regions were masked with the white rectangles. The intensity was subsequently subtracted by the averaged background intensity. The enhancement factor mapping in Figure S5(b) was calculated by the ratio between the intensity and the averaged intensity on flat gold regions masked with the red rectangles in Figure S5(a). In Figure S5(b), the highest UCL enhancement distributes around the periods

from 900 nm to 920 nm. This was in a good agreement with the UCL enhancement distribution on the grating matrix measured with our experimental setup.

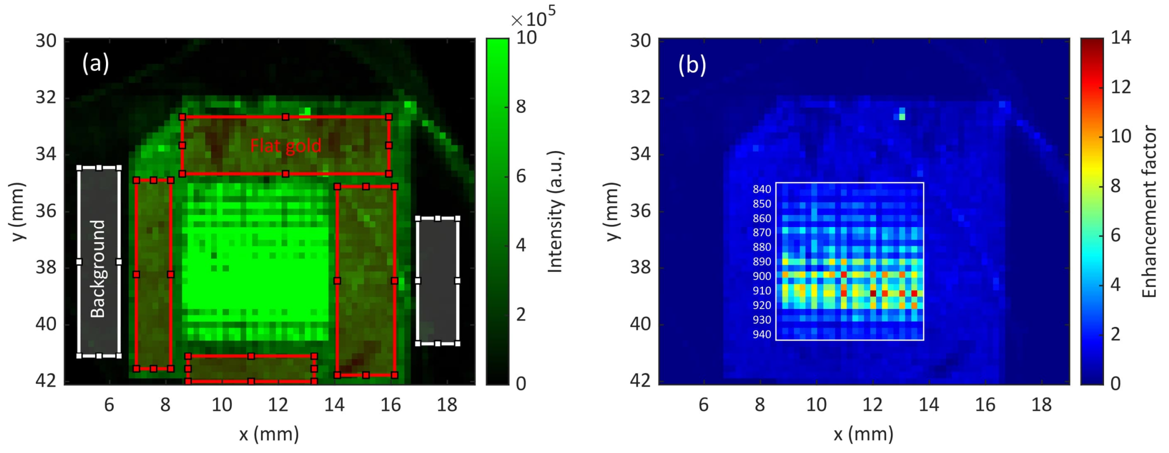

Figure S5: (a) UCL measurement with a commercial reader (Labrox Oy, Turku, Finland). The red and white rectangles indicate the flat gold and the background, respectively. The scanning area is 12×15 mm^2 with a scanning step of 0.2 mm. (b) Enhancement factor map of the grating matrix. The matrix is blurry due to the large scanning step.

References

[1] W. Zhao, X. Tian, Z. Fang, *et al.*, "Engineering single-molecule fluorescence with asymmetric nano-antennas," *Light Sci. Appl.*, vol. 10, no. 1, pp. 1–9, 2021.
[2] M. D. Wisser, S. Fischer, C. Siefe, A. P. Alivisatos, A. Salleo, and J. A. Dionne, "Improving quantum yield of upconverting nanoparticles in aqueous media via emission sensitization," *Nano Lett.*, vol. 18, no. 4, pp. 2689–2695, 2018.
